# Supplementary material for: Artificial intelligence for contextual well-being: Protocol for an exploratory sequential mixed methods study with medical students as a social microcosm
Source: PLoS One. 2025 May 28;20(5):e0321426. doi: 10.1371/journal.pone.0321426 (PMC12118909; doi:10.1371/journal.pone.0321426)
Supplement: S1 Appendix — (DOCX) [file pone.0321426.s001.docx]

Interview Instruction

As a semi-structured interview, the interviewer’s questions are informed by the systematic integrative review. These questions are designed around the initially identified themes from the review, with core themes selected and organised along a four-layer literatures gap (see fig below)

- Layer 1: Explores participants’ awareness of the themes.
- Layer 2: Examines participants’ actions in response to the awareness identified in Layer 1.
- Layer 3: Investigates how participants make decisions based on the actions described in Layer 1/Layer 2.
- Layer 4: Focuses on the intention for the themes, exploring how AI can facilitate these themes within a defined context—a well-designed AI infrastructure system.


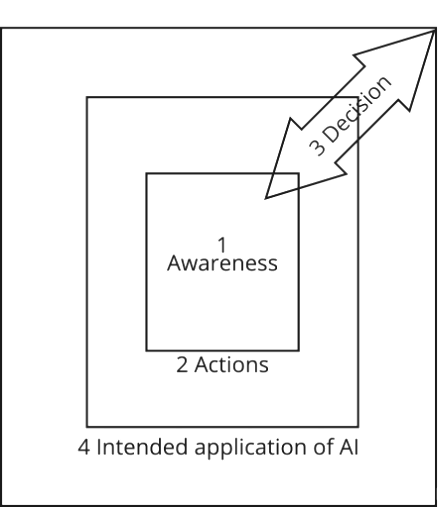


# Section 1 Human Centred

## Introduction

**Icebreaker 1**

Briefly introduce the intention of this interview. After a brief introduction of oneself, I will ask the participant not to reveal any highly personal or sensitive information, as well as no identifiable information about themselves or others.

This interview will last for approximately 60-90 minutes. I will start recording after you consent. Anything we discuss will remain confidential and your identity will remain anonymous in any publications or reports. Finally, you can stop the interview at any point if you wish. Do you have any questions for me before we get started?

**Opening Question**

Can you share a specific moment when you noticed significant changes in your behaviors, actions, mood, or physical health?

- What was happening during that time?
- How did you initially respond to these changes?
- What factors helped or hindered you in addressing those challenges?

**Follow-Up Exploration to Connect Identity, Resources, and Responsibility**

1. **Identity**:
   How did you perceive yourself during that time? Did your role, values, or personal beliefs influence how you approached the situation?
2. **Resources**:
   What resources, such as people, information, or support systems, were available to you? Were there any resources you felt were missing or inadequate?
3. **Responsibility**:
   Did you feel a sense of responsibility to act on these changes? What or who motivated (or demotivated) you to act?
4. Did you feel your role, identity greatly shape your resource, responsibility at that time?

**Connecting to Action (Seeking Help)**

- At what point did you realize you might need support? What factors made it easier or more difficult for you to seek help or act?
- How did you eventually address the issue, and what impact did that have on your behaviours, mood, or overall well-being afterward?

# Section 2 Artificial Intelligence

#### General Experience with AI

- In what contexts do you use AI?
- Have you interacted with any AI systems designed to improve well-being (e.g., apps, tools, platforms)? If so, can you describe your experience?
- Even if you haven’t used an AI system/tool specifically designed for well-being, can you think of any AI-driven tools or platforms that have positively impacted your health, mood, or learning and productivity so far?
- What features or functionalities of these systems stood out to you as helpful or engaging?

#### Perceived Nudges

- Have you noticed any behaviours or habits that have changed after interacting with AI tools? If yes, what do you think triggered those changes?
- Can you recall a specific instance where an AI feature influenced your decision-making or actions related to your well-being?
- Do you think that an AI system could help you make better decisions without taking away your ability to choose?
- How do you think an AI could help you act when you feel overwhelmed with your responsibilities?
- What type of action would you appreciate? Such as a gentle reminder to take a break, reprioritise tasks, focus on your health, or quality information?

#### Ethical and Emotional Impact

- How did using these AI tools make you feel? Did it feel like a supportive nudge, or did it ever feel pushy or controlling?
- Do you trust AI recommendations for well-being-related decisions? Why or why not?
- If an AI tool could analyse and verify information for you, how would you decide when to trust its recommendations? For example, health or daily?
- What features would increase or decrease your confidence in its guidance?

#### Potential for Improvement

- In your opinion, how can AI systems better support your well-being in the future?
- What kind of AI system would you find more effective or appealing as a medical student in university?
- How aware are you of the potential challenges surrounding AI governance, particularly in terms of Fairness, Accountability, Transparency, and Ethics that shape your community well-being?

#### Needs

Imagine an AI system designed to be responsible, ethical, and user focused. It respects your privacy, provides accurate and reliable insights, and aims to enhance your well-being without replacing your personal decision-making and providing you with more options. Can you imagine what kind of AI system could support your well-being?

## End

What does 'well-being literacy’ mean to you personally, how can universities support this in your opinion?

How would you rate your well-being literacy, on a scale from 1 to 5, with 1 being low and 5 being high?

I would then thank the student for his/her time. To conclude, I would inform the participants of the potential follow up through email (with consent), such that I can provide the individual a copy of the transcript of their interview for a member checking/follow up interview to occur.

## Technique to aid participants' response and understanding.

1. Encourage Narratives to Capture Complexity

Ask participants to describe specific incidents or experiences that encapsulate multiple aspects of context in the case the response wouldn't flow.

**Example:**

Can you walk me through a time when a challenging academic or personal situation forced you to draw on multiple resources or navigate cultural expectations?

2. Adaptive Question Flow

Interviews may involve situations where interviewees elaborate on certain dimensions without following the planned sequence, in particular section 1. Therefore, a checklist has been designed for the interviewer: if a dimension is triggered earlier in the conversation, it will be ticked off and skipped to avoid repetition.

3. Behavioural event interview technique

Behavioural event interview techniques will be incorporated for interviewees to describe specific past experiences or behaviours that demonstrate certain skills or competencies.

###

### **Interview Dimension Checklist (For Interviewer)**

### **Time and Space**

- Changes in life (past year)
- Impact of time and space on daily life (home, school, work, virtual)
- Past experiences influencing future approach
- Time management and environment handling

#### **Culture, People, and Interaction**

- Influence of people around (friends, family, classmates)
- Support from specific people or groups
- Cultural influences on handling challenges
- Connection with others (similar or different backgrounds)

#### **Information**

- Source of advice or guidance during uncertainty
- Trustworthiness of advice/information
- Experience of feeling stuck due to lack of information
- Steps taken to find reliable information

#### **Motions: State of Feeling, Body, and Behaviours**

- First signs of feeling off (emotionally/physically)
- Responses to emotional or physical changes
- Routines that help maintain balance
- Actions taken when balance is disrupted

#### **Identities**

**Biological Identity**

- Influence of gender on decision-making and behaviour
- Gender’s role in feeling supported or challenged

**Self-Identity**

- View of self beyond student, friend, or professional roles
- Identity's role in handling challenges

**Spiritual Identity**

- Influence of personal beliefs/values on decision-making
- Use of practices like reflection or meditation for clarity

**Cultural Identity**

- Impact of cultural background on decisions and challenges
- Adapting to cultural norms vs staying true to own practices

**Social Identity**

- Role in groups (school, family, etc.) and its influence on well-being
- Feeling of support or pressure from social groups

#### **Responsibilities**

- Most important responsibilities right now
- Feelings of overwhelm or manageable responsibilities
- Desire to delegate or share responsibilities
- Adjusting roles or expectations to ease responsibilities

#### **Resources**

- Definition of Resources
- Key resources relied on during tough times
- Accessibility and ease of accessing resources
- Connection between identity, responsibilities, and resources
- The balance between resources and identity-related responsibilities
